# Portal vein aneurysm combined with intrahepatic portosystemic venous shunt diagnosed by multimodal imaging techniques: A case report

**DOI:** 10.1097/MD.0000000000030475

**Published:** 2022-09-16

**Authors:** Xin Liu, Bingmu Liu, Liqun Guo

**Affiliations:** a Department of Ultrasonography, The First Central Hospital of Baoding, Baoding, Hebei Province, China; b Interventional radiology, The First Central Hospital of Baoding, Baoding, Hebei Province, China.

**Keywords:** case report, diagnosis, imaging, intrahepatic portosystemic venous shunt, portal vein aneurysm

## Abstract

Portal vein aneurysms (PVA) and intrahepatic portosystemic venous shunts are rarely diagnosed clinically.

**Patient Concerns:**

A 75-year-old female was admitted to our hospital for evaluation of significant weight loss, diabetes, and an irregularly shaped cystic lesion in the left lateral lobe of the liver.

**Diagnosis:**

The patient was diagnosed with a portal vein aneurysm combined with an intrahepatic portosystemic venous shunt using multiple imaging techniques.

**Interventions:**

The patient had no relevant clinical symptoms of PVA with concurrent intrahepatic portosystemic venous shunt; hence, no interventions were performed. Ultrasonography was suggested to be performed every 3 months.

**Outcomes:**

The patient did not visit the hospital after discharge; however, 4 telephonic follow-up evaluations showed that the patient was well.

**Lessons:**

Multimodal imaging techniques should be used to evaluate the source of blood flow, presence or absence of shunts, and the course, number, and location of the shunts to prevent misdiagnosis of this disease.

## 1. Introduction

Portal vein aneurysm (PVA) is an unusual form of portal vein vasodilatation with an incidence of 0.06%.^[1]^ Patients with PVA are usually asymptomatic. Indeed, most patients with PVA are detected incidentally by computed tomography (CT), magnetic resonance imaging (MRI), and ultrasound.^[1,2]^ Portal hypertension is the leading cause of acquired PVA.^[1]^ Conservative management or surgery is recommended based on the patient’s underlying risks and symptoms.^[1,2]^ A congenital portosystemic venous shunt is a rare type of congenital dysplasia in which the intrahepatic portal vein communicates with a systemic vein through an abnormal intrahepatic venous channel, usually along with secondary complications such as hypoxia and focal intrahepatic lesions.^[3]^ The characteristics and natural course of intrahepatic malformations vary in these patients.^[4]^ A spontaneous intrahepatic portosystemic venous shunt is also rare, with an extremely low incidence in adults.^[5]^ Herein, we report a patient with PVA and intrahepatic portosystemic venous shunt, diagnosed using multimodal imaging techniques.

## 2. Case report

A 75-year-old female was admitted to our hospital for evaluation of significant weight loss and diabetes on October 13, 2020. She was prescribed the following medications before admission: metformin (0.5 g twice a day), acarbose (50 mg 3 times a day), and repaglinide (1 mg twice a day). The metformin dose was increased to 1 g/day without medical advice 6 weeks before admission. She had coronary heart disease and was prescribed the following medications: isosorbide nitrate tablets, 5 mg in the morning, 10 mg in the afternoon, and 5 mg in the evening; 10 capsules of Danshen dripping pills, 3 times a day; and atorvastatin, 10 mg once in the evening. After admission, the physician considered that the weight loss might be related to metformin; therefore, metformin was discontinued. The acarbose capsules and repaglinide tablets were discontinued due to blood glucose level fluctuations, and a subcutaneous injection of insulin was used to lower the blood glucose level. After admission, the pre-hospital medication regimen for coronary heart disease was continued.

**Figure 1.**
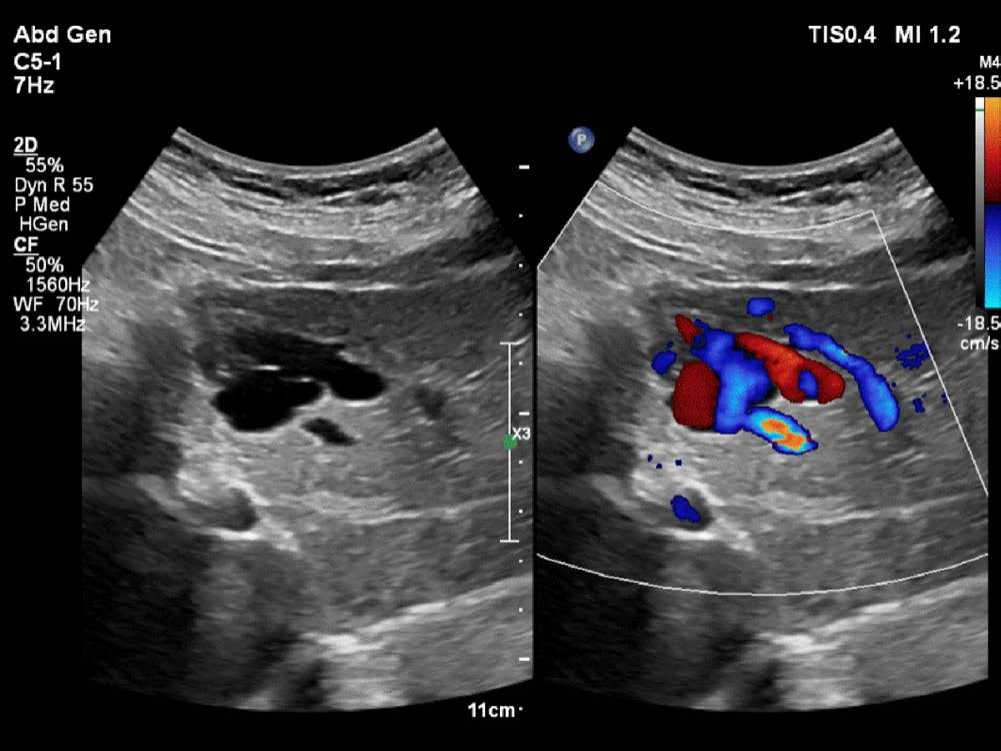
A color Doppler showed a cystic hepatic lesion with blood flowing through a channel to the medial branch of the left hepatic vein.

**Figure 2.**
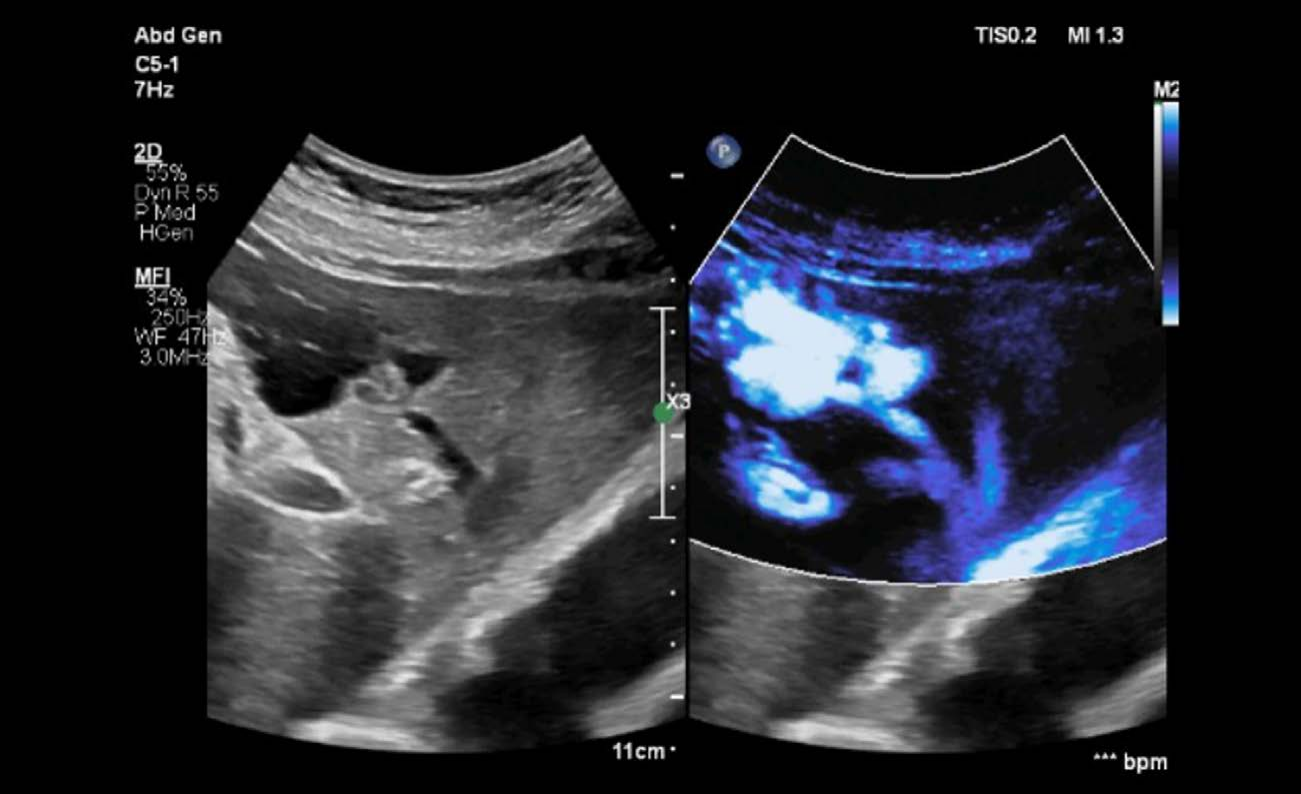
Microvascular imaging demonstrated the passage between the cystic lesion and the left hepatic vein.

The patient had no history of hepatitis, trauma, or liver surgery. She denied having a family history of diabetes, hereditary or infectious diseases. A cyst was previously diagnosed in the left lobe of the liver using ultrasonography. CT examination of the upper abdomen revealed a round, low-density shadow in the left lobe of the liver with clear borders. A routine ultrasound examination post-admission showed an irregularly shaped cystic lesion in the left lateral lobe of the liver, which was approximately 4.9 × 3.3 × 2.4 cm in size. Red and blue vortex signals were observed in the cystic lesion on color Doppler flow imaging (CDFI)(Fig. 1). Further observations showed that the inner diameters of the left portal and hepatic veins had widened. The cystic lesion was closely related to the sagittal part of the liver and continued through the left branch of the portal vein; a channel was shown to be connected to the medial branch of the left hepatic vein. The channel predominantly showed blue-colored blood flow signals under CDFI, suggestive of a venous spectrum with a flow rate of approximately 50 cm/s. The 2-dimensional ultrasound and color Doppler ultrasound findings were as follows: blood flow signals were noted in the cystic lesion, which negated the previous diagnosis of liver cysts; one end of the cystic lesion was connected to the sagittal part of the portal vein, and the other end was connected to the left medial branch of the hepatic vein. Further microvascular imaging confirmed that the blood flow in the cystic echo was connected to the left branch of the portal vein and the medial branch of the left hepatic vein (Fig. 2).

**Figure 3.**
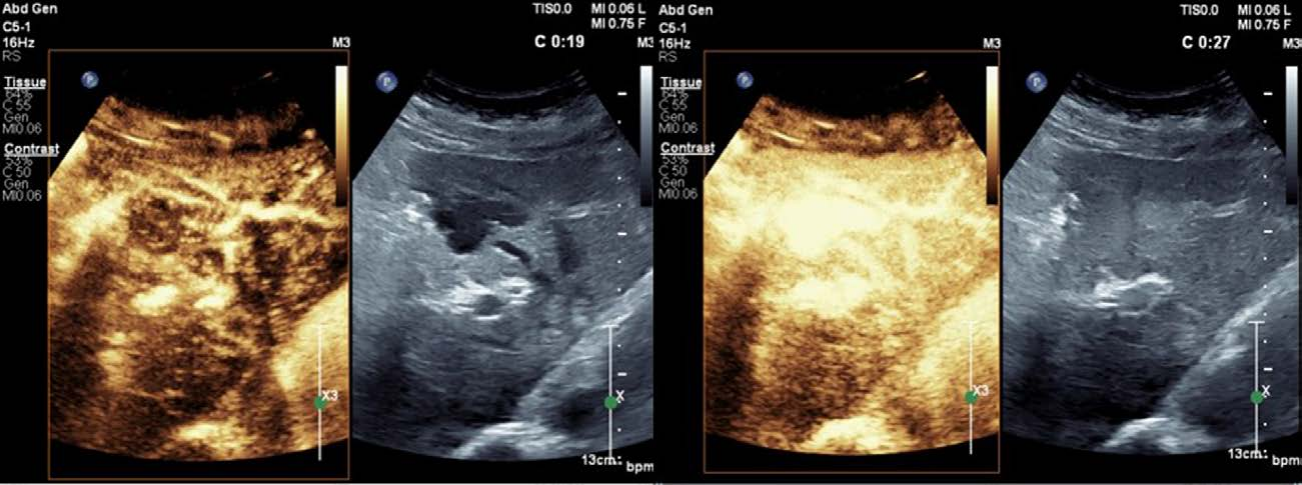
In both the portal vein stage and the delayed stage, the cystic lesion was found to communicate with multiple channels in the left hepatic vein.

Contrast-enhanced ultrasound was performed to better delineate the lesions and determine the patient’s condition. The results showed that the intrahepatic cystic lesions did not enhance during the arterial phase. At the portal vein stage (22 s), enhancement began to appear around the lesion. In both the portal vein and delayed phases, multiple channels connected the cystic lesion with the internal and external branches of the left hepatic vein (Fig. 3). The results suggested that the branch of the portal and hepatic veins communicated through a tubular or tumor-like structure during the portal vein phase, and the hepatic vein was enhanced early and nonuniformly. A portosystemic shunt can show transient, non-specific, uniform relative enhancement during the portal vein and delayed phases of contrast-enhanced ultrasonography and may lead to increased blood flow in the shunt portal vein, resulting in reduced blood flow to the normal liver parenchyma surrounding the lesion.^[6]^ The findings in this patient were similar to those seen in portosystemic shunts. MRI and further contrast-enhanced examination of the upper abdomen revealed a round-shaped abnormal signal shadow in the left lateral lobe of the liver. The shadow measured approximately 4.5 × 2.0 × 3.0 cm, showing uneven long T1 and T2 signals, and low signals on diffusion-weighted imaging. The arterial phase enhanced slightly after injection of the contrast agent. A tortuous and thickened vascular shadow was found during the portal phase, with a signal intensity after contrast enhancement similar to the portal vein. The lesions showed communication with the left hepatic vein during the balanced phase and merged with the inferior vena cava. MRI results suggested an abnormal shadow in the left lobe of the liver, a portosystemic shunt, and PVA formation.

The final diagnosis was PVA combined with an intrahepatic portosystemic venous shunt. Because the patient had no relevant clinical symptoms because of the PVA and intrahepatic portosystemic venous shunt, the physician did not take any measures but recommended close follow-up. Interventional surgery was recommended if the patient experienced pain or discomfort. The patient was subsequently treated for diabetes and coronary heart disease after discharge from the hospital on November 1, 2020, without intervention for PVA and intrahepatic portosystemic venous shunt. Subcutaneous insulin treatment for diabetes was continued. The pre-hospital medication regimen for coronary heart disease was continued after discharge. The physician instructed the patient to undergo an ultrasound every 3 months; however, the patient did not return for the ultrasound examination because of her old age and limited mobility. The patient was asymptomatic during 4 telephonic follow-up evaluations. The treatment plan was to continue closely observing her condition. This study was approved by the ethics committee of the First Central Hospital of Baoding. The patient provided written informed consent for this study.

## 3. Discussion

Portal vein tumors combined with a congenital portosystemic venous shunt are extremely rare. This case was diagnosed as PVA with a portosystemic venous shunt using multimodal imaging.

A single imaging modality has limitations in diagnosing diseases, especially rare and complex ones.^[7]^ Several factors influenced the diagnosis in this case, such as the nature of the internal blood flow (arterial or venous), source of blood flow, presence or absence of shunts, and the course, number, and location of shunts. Hence, we used multimodal imaging to confirm the diagnostic results in this case. Color Doppler ultrasound can macroscopically detect abnormal tortuous blood vessels or irregular cystic lesions in the liver.^[8]^ In the case of hepatic cystic lesions, an ultrasound can easily and quickly display the internal blood flow, identify cysts and arteriovenous aneurysms, and determine whether arterial or venous blood flow is present.^[9]^ In this patient, an ultrasound examination after admission showed an irregularly shaped cystic lesion in the left lateral lobe of the liver, in which red and blue eddy current signals were seen. The cystic lesion was closely related to the sagittal part of the liver and continued with the left branch of the portal vein, a channel communicating with the medial branch of the left hepatic vein, and was dominated by a blue festoon blood flow signal, suggestive of a vein-like signal spectrum. A previous study showed that portal hypertension is the leading cause of acquired PVA.^[1]^ Iwao et al found that a portal venous velocity of 13 cm/s, hepatic arterial pulsatility index of 1.1, and liver vascular index of 12 cm/s were the best cut-off values for the ultrasound diagnosis of portal hypertension.^[10]^ Pulsed-wave Doppler can not only evaluate the direction of blood flow but can also estimate the flow rate to determine the diversion rate.^[11]^ In our case, the connection between the portal and hepatic veins was clearly shown by color Doppler ultrasound, which showed that the flow velocity of the shunt vessels in the portal vein increased with the resistance index, and turbulence was visible at the connection site. Microvascular imaging using the new algorithm can provide a differential diagnosis for liver lesions by reducing the influence of fluctuating clutter and eventually increasing the rate of blood flow signal detection at low speeds.^[12]^ Multiple channels from the cystic lesion were observed connecting different branches of the left hepatic vein, and low-velocity blood vessels were also discovered by microvascular imaging in our patient, which could not be displayed by the conventional color Doppler ultrasound. On a contrast-enhanced ultrasound scan, a branch of the portal vein and the hepatic vein could be seen communicating through a tubular or tumor-like structure during the portal venous phase, with early and heterogeneous enhancement in the hepatic vein, and transient, non-specific, and homogeneous relative enhancement during the portal venous and delayed phases. Congenital portosystemic shunt results in accelerated portal-hepatic circulation time on contrast-enhanced ultrasound scans. In addition, contrast-enhanced ultrasound technology effectively identifies portal vein tumors.^[13]^ Using this technology, the time of arrival of the contrast agent to the hepatic artery, portal vein, and hepatic vein (HVAT) were recorded. The time interval for the arrival of the contrast agent from the hepatic artery to the hepatic vein (HA-HVAT) and the transit time for the contrast agent to reach the hepatic vein from the portal vein (PV-HVTT) was calculated. A congenital portal-systemic venous shunt can accelerate the circulation time of the portal and hepatic veins and shorten the HVAT, HA-HVAT, and PV-HVTT.^[3,4]^ Some patients can be diagnosed with a congenital portal-systemic shunt through prolongation of the hepatic vein imaging phase when no clear connection lesions are seen.^[14]^ Multimodal imaging techniques helped perform a detailed analysis of the case, which led to an accurate diagnosis of the disease. Multimodal imaging examinations are significant for early clinical detection, diagnosis, and treatment of PVA and portosystemic venous shunt.

A portal vein diameter > 1.9 cm in cirrhotic patients or 1.5 cm in normal livers is defined as PVA.^[1]^ PVAs are easily misdiagnosed as hepatic cysts, intrahepatic bile duct dilatation, and intrahepatic arteriovenous fistulas.^[1]^ Therefore, during the imaging process, it is necessary to continuously scan multiple sections, carefully analyze the blood flow spectrum, accurately determine the source of blood supply and classification, and avoid misdiagnosis.^[15]^ Most patients with a PVA undergo conservative management with close follow-up evaluations.^[1,2,16]^ Due to the long-term presence of an abnormal anatomic structure typical of a portosystemic venous shunt, patients are at risk of significant medical complications such as bleeding, pulmonary hypertension, and hepatic encephalopathy.^[3]^ If the shunt causes symptoms, an active management approach such as ligation or embolization should be adopted.^[3]^ Rigorous and detailed imaging examinations are also needed to evaluate the anatomic characteristics and location of the shunt.^[7–15]^ Multimodal imaging techniques, such as color Doppler ultrasound, micro-flow imaging technology, contrast-enhanced ultrasound examination, and magnetic resonance imaging (MRI), have become effective tools for diagnosing portosystemic venous shunts. In addition, 3-dimensional ultrasound research can be added to provide a basis for the differential diagnosis of portosystemic venous shunt.^[17]^ If the blood flow through the shunt is small, clinical observation may be performed, and only symptomatic treatment may be administered when required. If the blood flow through the shunt is significant, further interventions such as surgery or interventional therapy can be used.

## 4. Conclusion

In conclusion, this case demonstrates that sonographers should evaluate the patient’s medical history for indications of PVA and intrahepatic portosystemic venous shunt while considering the differential diagnoses for hepatic cystic lesions. During the examination, multiple sections were scanned to analyze the source of blood flow, presence or absence of shunts, and the path, number, and location of shunts to prevent misdiagnosis and formulate a reasonable treatment plan for patients.

The lessons from this case were, first, ultrasound can easily and quickly identify hepatic cysts and arteriovenous aneurysms and assess the internal blood flow. The nature of the blood flow can be determined to be arterial or venous. Second, timely application of contrast-enhanced ultrasound and microvascular imaging techniques can determine the source of blood flow, the presence or absence of a shunt, and the shunt’s course, number, and location.
